# Improvement of Emulsion Stability and Plugging Performance of Nanopores Using Modified Polystyrene Nanoparticles in Invert Emulsion Drilling Fluids

**DOI:** 10.3389/fchem.2022.890478

**Published:** 2022-05-30

**Authors:** Xianbin Huang, Xu Meng, Leping Wu, Chongyang Gao, Kaihe Lv, Baolu Sun

**Affiliations:** ^1^ Key Laboratory of Unconventional Oil & Gas Development (China University of Petroleum (East China)), Ministry of Education, Qingdao, China; ^2^ School of Petroleum Engineering, China University of Petroleum (East China), Qingdao, China; ^3^ CNPC Bohai Drilling Engineering Company Limited, Tianjin, China; ^4^ Fuyu Oil Production Plant of Jilin Oilfield Company, Songyuan, China

**Keywords:** oil-based drilling fluids, nano-plugging agents, microporous membrane, emulsion stability, nano-material

## Abstract

Drilling fluid invasion and pressure transmission caused by the development of micropores and fractures in shale oil and gas formations are the major factors contributing to wellbore instability during drilling using oil-based drilling fluids (OBFs). In this study, a modified polystyrene latex (MPL) material was synthesized through emulsion polymerization and was characterized using Fourier transform infrared spectroscopy (FTIR), thermogravimetric analysis (TGA), particle size analysis, scanning electron microscopy (SEM) observations, and contact angle testing. The influence of the MPL on the stability of a water-in-oil emulsion was analyzed *via* sedimentation observations and electrical stability tests. The effects of the MPL on the plugging mechanism of white oil and water-in-oil emulsions were evaluated using 0.1–1.0 μm micro-porous filtration films. The experimental results revealed that the MPL has a favorable thermal stability, with an initial thermal decomposition temperature of 363°C, a median particle size (D50) of 233 nm, and a three-phase contact angle of 103.5°. The MPL can enhance the sedimentation stability of an emulsion to a considerable extent and can improve the electrical stability (ES) of the emulsion, which is conducive to the stability of OBFs. Due to the deformability of the MPL, it has a wide range of adaptations for micro-scale pores and fractures. In both the white oil and water-in-oil emulsions, the MPL can reduce the filtration loss through microporous membranes with pore sizes of 0.1–1.0 μm to within 10 ml. This paper details the methodology of the synthesis of nanomaterials that can effectively plug a formation’s nanopores and fractures; thereby, stabilizing OBFs.

## Introduction

With the increasing shortage of conventional oil and gas resources, unconventional sources, such as resources derived from shale oil and gas, have become important replacements worldwide. However, the process of drilling shale formations for oil and gas is prone to challenges such as wellbore instability (Zeng et al., 2021; Zhang et al., 2022), which significantly prolongs the drilling cycle and adversely impacts the economic viability. Compared with water-based drilling fluids (WBFs), inverse emulsion oil-based drilling fluids (OBFs) have unique advantages in shale oil and gas drilling and are widely used (Guo et al., 2012; Li et al., 2014). The main reason for their versatility is that OBFs have strong inhibitory properties (Gao et al., 2021), which are necessary to maintain wellbore stability. Nonetheless, due to the complex geochemical properties of shale oil and gas formations, the use of OBFs do not solve the problems associated with wellbore instability in shale formations under complex conditions.

Currently, research on wellbore instability in shale oil and gas formations has mainly focused on shale hydration inhibition mechanisms (Huang et al., 2018a, 2018b; Du et al., 2020; Ma et al., 2021). Due to the increased usage of OBFs, research into the controls of shale wellbore collapse has been extended to studies of the micro-structures of shale and other formations (Arif et al., 2021; Tian et al., 2021). The infiltration and pressure transmission of drilling fluids due to the development of micro-pores and fractures in shale formations, most of which are nanoscale (Chen et al., 2021), are important factors leading to the instability and collapse of wellbores.

A great deal of research has been conducted on nano-plugging agents in WBFs, which include inorganic nanomaterials such as nanosilica (Shadizadeh et al., 2015) and polymer nanospheres such as polymer latex(Liu et al., 2015). However, less research has been conducted on the applicability of nano-plugging agents in OBFs. Li et al. (Li et al., 2020) synthesized a styrene butadiene resin/nano-SiO_2_ (SBR/SiO_2_) latex composite as a nano-plugging agent for OBFs. SBR/SiO_2_ can effectively reduce the filter loss and pressure transmission, stabilizing the wellbore. Geng et al. (Geng et al., 2021) synthesized a surface-modified nanoscale polystyrene material as a plugging agent for OBFs, which can improve the contact angle of the mineral oil on the rock surfaces, reduce the amount of imbibition of the oil into the rock, and maintain the wellbore stability. Xie et al. (Xie et al., 2015) synthesized a hyperbranched polyamine (HBPA) material with an average particle size of 36.7 nm. HBPA can decrease the permeability of artificial cores and slightly increase the emulsion stability of OBFs.

Colloidal particles form a membrane at the oil-water interface, which prevents droplet aggregation and stabilizes the emulsion (Ramsden, 1904; Pickering, 1907). This type of emulsion, which is known as the Pickering emulsion, consists of solid particles acting in place of, or with, traditional surfactants to stabilize the emulsification. Furthermore, the contact angle of the solid particles at the oil-water interface is a critical parameter. Most studies have shown that when the contact angle (θ) of the particles at the oil-water interface is slightly greater than 90°, most of the particle surfaces will be in the oil phase, which makes the formation of water-in-oil (W/O) emulsions more likely. In contrast, when θ is slightly less than 90° at the oil-water interface, most of the particle surfaces will be in the water phase, which makes it more likely that an oil-in-water (O/W) emulsion will form (Melle et al., 2005). In addition, it has been reported that nanoparticles with incompatible wettability may adversely affect the emulsion stability (Javadian and Sadrpoor, 2020; Jia et al., 2020). However, despite the fact that the emulsion stability is an important measure of the performance of an oil-based drilling fluid, few studies have been conducted on the nano-plugging agents with good emulsion stabilizing capability.

In this study, a modified polystyrene latex (MPL) material capable of stabilizing water-in-oil (inverse) emulsions was synthesized *via* emulsion polymerization, and its performance as a nano-plugging agent for OBFs was assessed.

## Materials and Methodology

### Materials

The styrene (St, 99.5 wt%) and stearyl methacrylate (SMA, 99 wt%) were purchased from the Shanghai MacLean Biochemical Technology company. The alkylphenol ether sulfosuccinate sodium salt (OS, 40 wt%) was obtained from the Shandong Yousuo Chemical Technology company. The ammonium persulfate (APS, 98 wt%), calcium oxide (96 wt%) and Span 80 were bought from the Sinopharm Chemical Reagent company, and the #5 white oil was obtained from the Shenzhen ZRT Chemical company. Polystyrene nanosphere latex (90 nm, 30 wt%) was synthesized in the laboratory.The industrial-grade organic bentonite (organoclay) was provided by the Bohai Drilling Company. The polytetrafluoroethylene (PTFE) microporous membranes were purchased from the Delv Science and Technology Company.

### Preparation of Modified Polystyrene Nano-Latex

The size and shape of the formation pores encountered during the drilling process are diverse. Rigid plugging materials are hard to deform and difficult for to adapt to these pore. The flexible plugging materials are beneficial to improve the plugging effect of drilling fluid by their deformation. At the same time, the plugging materials also need to have a certain strength to withstand the bottom hole pressure. Therefore, we synthesized a flexible polymer with a certain strength by combining the rigid monomer styrene (St) and the flexible monomer stearyl methacrylate (SMA).

MPL was synthesized by emulsion polymerization. First, the monomers (St and SMA) were washed with a 5 wt% aqueous NaOH solution to remove the polymerization inhibitor prior to use. Then, they were repeatedly rinsed with deionized water until the pH was equal to 7 (neutral). Finally, they were dehydrated with anhydrous sodium sulfate. Subsequently, 30 g of St, 8.5 g of SMA, and 3 g of emulsifier OS were added to 90 ml of water and were mixed at a high speed (6,000 rpm) using a shear emulsifier for 10 min to form a stable oil-in-water emulsion. Then, the emulsion was added to a 250 ml round-bottom three-neck flask equipped with a stirring device and a thermometer, and the stirring speed was set to 350 rpm. The temperature of the water bath was set to 80°C. When the temperature of the liquid in the flask reached 80°C, 3 ml of 5 wt% APS aqueous solution were added to initiate the reaction. The reaction was terminated after heating for 4 h, and the modified polystyrene nano-emulsion was obtained. The MPL mentioned in this article refers to a modified polystyrene microsphere aqueous dispersion with a solid content of 30%.

### Characterization

#### Fourier Transform Infrared Spectroscopy and Thermogravimetric Analysis

The MPL was repeatedly washed and centrifuged (10,000 rpm) with ethanol and deionized water, after which the emulsion was dried in an oven at 105°C for 24 h to obtain solid samples. The MPL solid sample was then homogenized into a fine powder for Fourier transform infrared spectroscopy (FTIR) and thermogravimetric analysis (TGA).

The 1–2 mg of fine MPL powder and 200 mg KBr were further ground and mixed uniformly, and then, the mixture was pressed into tablets. The infrared absorption of the MPL was measured in the range of 4000–400 cm^−1^ using a Fourier transform infrared spectrometer (IRTRacer-100, Shimadzu, Japan).

Approximately 10 mg of MPL sample was loaded in an open ceramic crucible. The thermogravimetric curve of the MPL was generated using a thermogravimetric analyzer (Model TGA2, METTLER TOLEDO, Switzerland) with a range of 40–600°C in a nitrogen atmosphere at a heating rate of 10°C/min.

#### Particle Size Analysis and Micromorphology

The MPL was diluted to 0.25% with deionized water. The particle size distribution at room temperature was measured using a laser nanoparticle sizer (Zetasizer Nano Model ZS90, Malvern, UK).

A drop of 0.25% MPL water dispersion was set on conductive carbon tape and was dried using an infrared heating lamp. The microscopic morphology of the MPL was observed using a scanning electron microscope (SEM) (Nova NanoSEM 450, FEI, USA).

#### Contact Angle Testing

The purified and dried MPL was pressed using a hydraulic device for 5 min under 10 MPa at room temperature to produce a thin film with a smooth surface. The contact angle of the deionized water on the surface of the MPL film, under open air conditions, was measured using an optical contact angle meter (model OCA25, Dataphysics, Germany).

**FIGURE 1:**
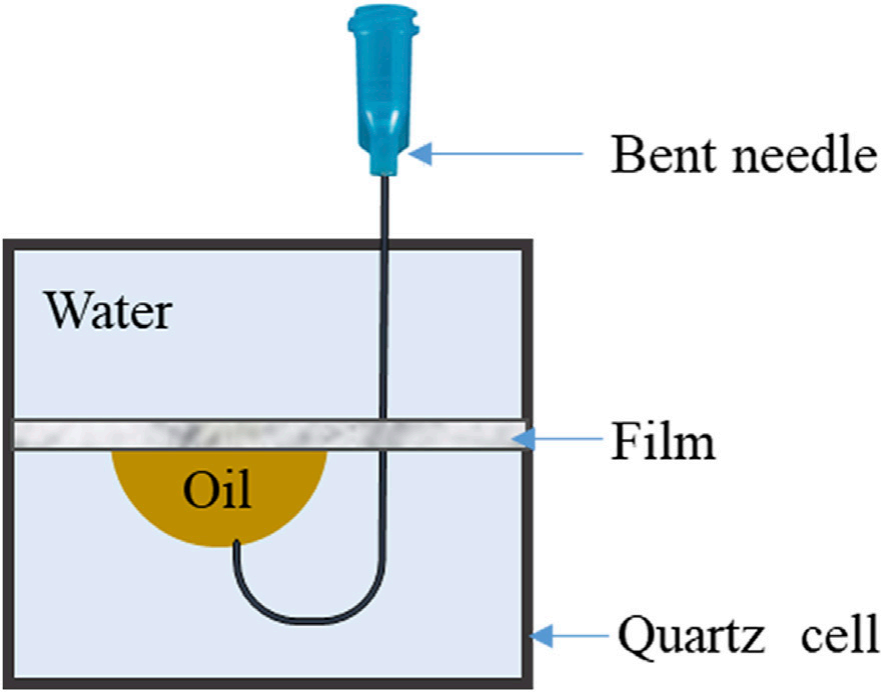
Schematic diagram showing the contact angle test method for the MPL film with white oil in an aqueous environment.

Subsequently, another MPL film was fixed in a sample cell filled with water, and a curved needle was used to place a drop of white oil on the lower surface of the MPL film. After creating a stable droplet shape, the contact angle between the MPL film and the white oil in a water environment was measured and tested using the method illustrated in Figure 1.

### Stability of Emulsions

#### Formulation of the Emulsion

In the formulation of OBFs, the most common oil-to-water volume ratio used is 80:20. In this experiment, a water-in-oil (inverse) emulsion with an oil-water volume ratio of 80:20 was formulated to study the effect of the MPL on the emulsion stability.

White oil (240 ml) and Span 80 (3 g) were added to five 500 ml beakers and the mixtures were stirred to promote the dissolution of the Span 80. Varying quantities of the MPL were added to the five beakers. The quantities of MPL added to the five beakers were 0 g, 1.5 g (0.5%), 3.0 g (1.0%), 6.0 g (2.0%), and 9.0 g (3.0%). The mixture in each beaker was stirred at a high speed (10,000 rpm) using a shear emulsifier. After 5 min, 60 ml of deionized water were added to the mixtures and the stirring was resumed for 20 min.

#### Emulsion Stability

For each of the prepared emulsions, 20 ml were placed in a stoppered colorimetric tube and left to stand. Pictures were taken at regular intervals to record the sedimentation.

The electrical stability (ES) was measured using an electrical stability meter (Model 23C, FANN, US). First, the ES values of the emulsions with different MPL concentrations were measured. After the tests, 4 g (2%) of organoclay were added to each 200 ml emulsion sample, and the ES values were tested again after another round of high-speed stirring for 20 min.

#### Particle Size of the Emulsion

The effect of the MPL on the particle size distribution of the emulsion was measured *via* focused beam reflectance measurement (FBRM, ParticleTrack G600, METTLER TOLEDO, Switzerland).

### Evaluation of the Plugging Performance

#### Preparation of Plugging Fluids

Two types of plugging fluids were prepared: MPL dispersions in white oil and MPL dispersions in an emulsion with an oil-water ratio of 80:20.

MPL dispersions in white oil: 1 wt/v% Span 80 (3 g) and MPL of different masses were added to 300 ml of white oil and were stirred at high speed (10,000 rpm) for 20 min to obtain MPL white oil dispersions.

MPL dispersions in an emulsion: To 300 ml of white oil, 1 wt/v% Span 80 and MPL of different masses were added. Using a shear emulsifier, the above mixture was stirred at the same speed (10,000 rpm) for 5 min, and 60 ml of deionized water were added. Following this addition, the stirring continued for an additional 20 min to obtain dispersions of the MPL in an inverse emulsion.

#### Plugging Tests

In the experiments, a polytetrafluoroethylene (PTFE) microporous membrane with a diameter of 9.0 cm was used. The pore sizes of the microporous membranes used as filtration mediums were 0.1, 0.3, 0.5, and 1.0 μm. An API filtrate meter (Model SD6A, Tongchun, Qingdao, China) was used to measure the filtration loss with time of the two plugging fluids with different MPL concentrations. The testing conditions of the filter loss experiment were 25°C at 0.7 MPa. In the experiments, a high-precision balance was used to record the change in the quantity of the filtrate in real time. By converting the mass into volume, the change in the filtrate volume with time was obtained. The density of the filtrate was calculated using a density of *ρ* = 0.818 g/cm^3^.

## Results and Discussion

### FTIR Analysis

**FIGURE 2:**
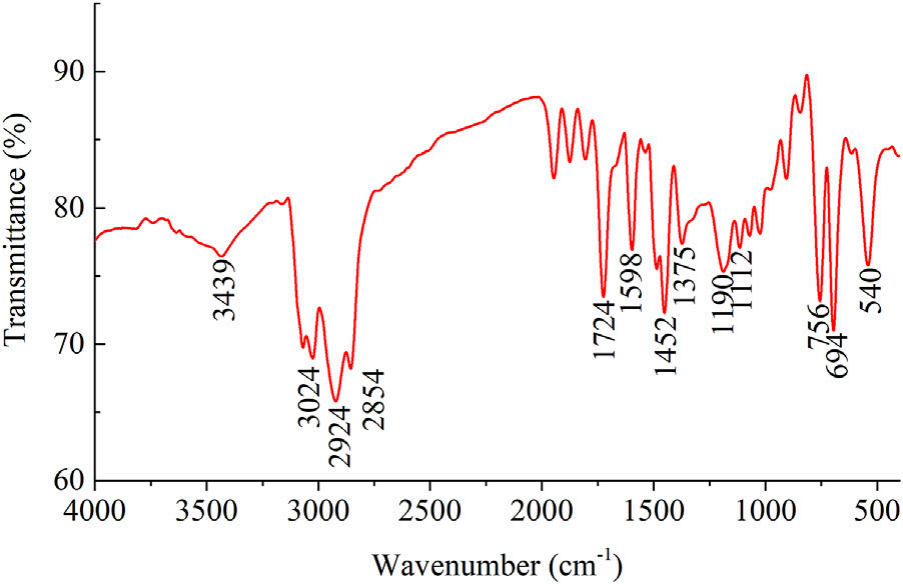
The fourier transform infrared (FTIR) spectrum of the powder sample of the modified polystyrene latex (MPL).

Figure 2 presents the infrared spectrum of MPL powder sample. O-H stretching vibration is primarily responsible for the peak at 3,439 cm^−1^. The 3,024 cm^−1^ at peak is due to the aromatic C-H stretching vibration coming from styrene (Huang et al., 2018b). The peaks at 2,924 cm^−1^ and 2,854 cm^−1^ are attributed to C-H stretching vibration of the CH_2_ and CH groups. The narrow but strong peak at 1,724 cm^−1^ is the result of C=O stretching vibration. In addition, the peak at 1375 cm^−1^ is caused by the bending vibration of C-H; and the peaks at 1,112 cm^−1^ and 1,190 cm^−1^ are the characteristic bands of C-O-C. The peaks at 694 cm^−1^ and 756 cm^−1^ indicate the presence of monosubstituted phenyl groups derived from the styrene. The peaks at 1,452 cm^−1^ and 1,598 cm^−1^ are typical benzene skeletal vibration peaks.

### Thermogravimetric Analysis

**FIGURE 3:**
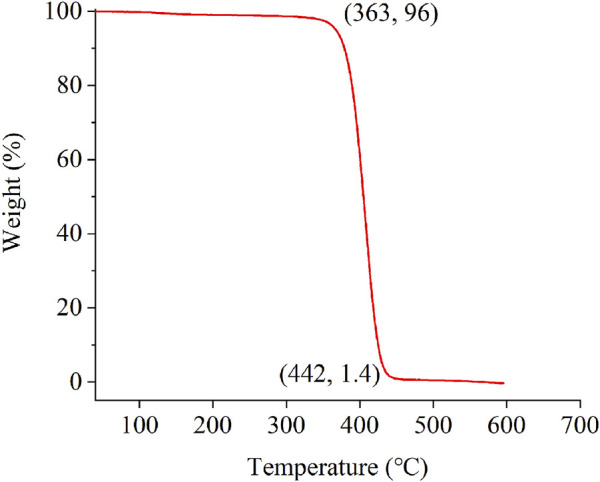
Thermogravimetric analysis curve of the powder sample of the modified polystyrene latex (MPL).

Figure 3 shows the TGA curve of the MPL. The decrease in the weight of the MPL can be roughly divided into three stages (Figure 3). The first stage of weight loss occurred from room temperature to 363°C, and the slope of the TGA curve remained almost unchanged. The weight loss rate at 363°C was only 4%, which was caused by the volatilization of the free and bound water in the MPL, indicating that the MPL powder had good thermal stability in the nitrogen environment. The second stage was 363–442°C. In this stage, the weight of the MPL decreased sharply, indicating that the MPL began to thermally degrade at a rapid rate. When the temperature reached 442°C, the mass of the MPL was only 1.4%, indicating that thorough thermal degradation had occurred. At temperatures of > 442°C (the third stage), the MPL approached the limit of its thermal degradation, and the TGA curve became stable. Consequently, the TGA experiments revealed that the initial decomposition temperature of the MPL in a nitrogen environment (363°C) was indicative of a higher thermal stability.

### Particle Size and Micromorphology

**FIGURE 4:**
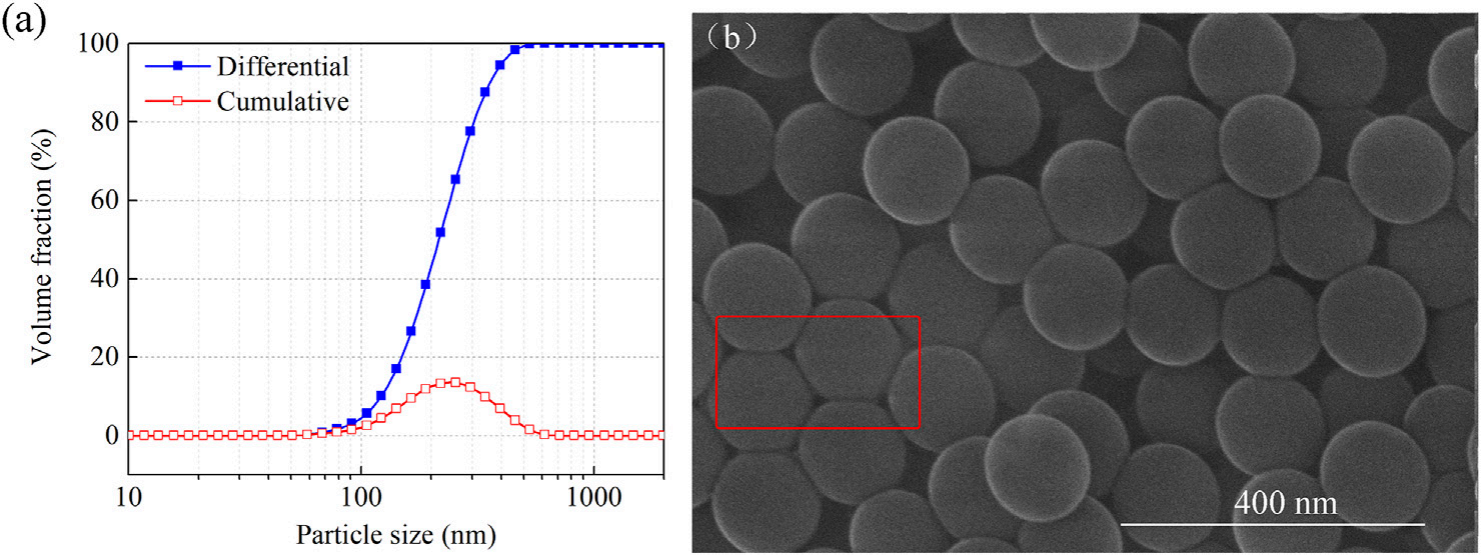
**(A)** Particle size distribution of MPL nanoparticles and **(B)** SEM image of MPL.

The particle size distribution of the MPL was measured. The experimental results are shown in Figure 4A. The particle size distribution of the MPL at room temperature exhibited a wide range (50–600 nm), with a median value (D50) of 233 nm. The scanning electron microscopy results revealed that the MPL particles were spherical and uniform in shape (Figure 4B). Due to accumulation, some of the particles also had a hexagonal shape (marked in Figure 4B), indicating that the MPL were flexible nanospheres and that the particles could be deformed. This is ultimately beneficial to plugging micro-scale pores and fractures in formations.

### Wettability Analysis

**FIGURE 5:**
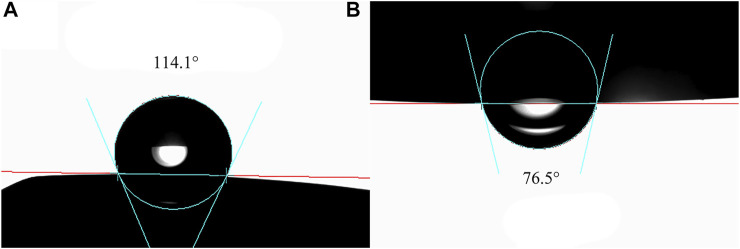
**(A)** Contact angle of deionized water on the surface of MPL film in an open air environment. **(B)** Contact angle of white oil on the surface of MPL thin film in an aqueous environment.

In the open atmosphere, mineral oil spread quickly along the surface of the MPL film with a contact angle of 0°. The contact angle of the deionized water on the surface of the MPL film was 114.1° (Figure 5A). This revealed that the MPL was more lipophilic than hydrophilic. It was difficult to accurately measure the contact angle of the water droplets on the surface of MPL films in oil environments. This is due to the fact that when the film was placed in the oil-dominant environment, the oil will become tightly adsorbed onto the surface of the film. As such, the water droplets will fail to dislodge the oil and spread on the surface of the film. More specifically, since the adsorption force between the water and the film was much weaker than the force between the oil and the film, the oil droplets were more capable of displacing the water and spreading over the surface of the MPL. Therefore, the contact angle of the oil droplets on the MPL surface was determined in an aqueous environment. The contact angle of the stabilized white oil on the MPL surface was 76.5° (Figure 5B), and the three-phase contact angle of the deionized water on the MPL surface was 103.5°.

### Emulsion Stability

**FIGURE 6:**
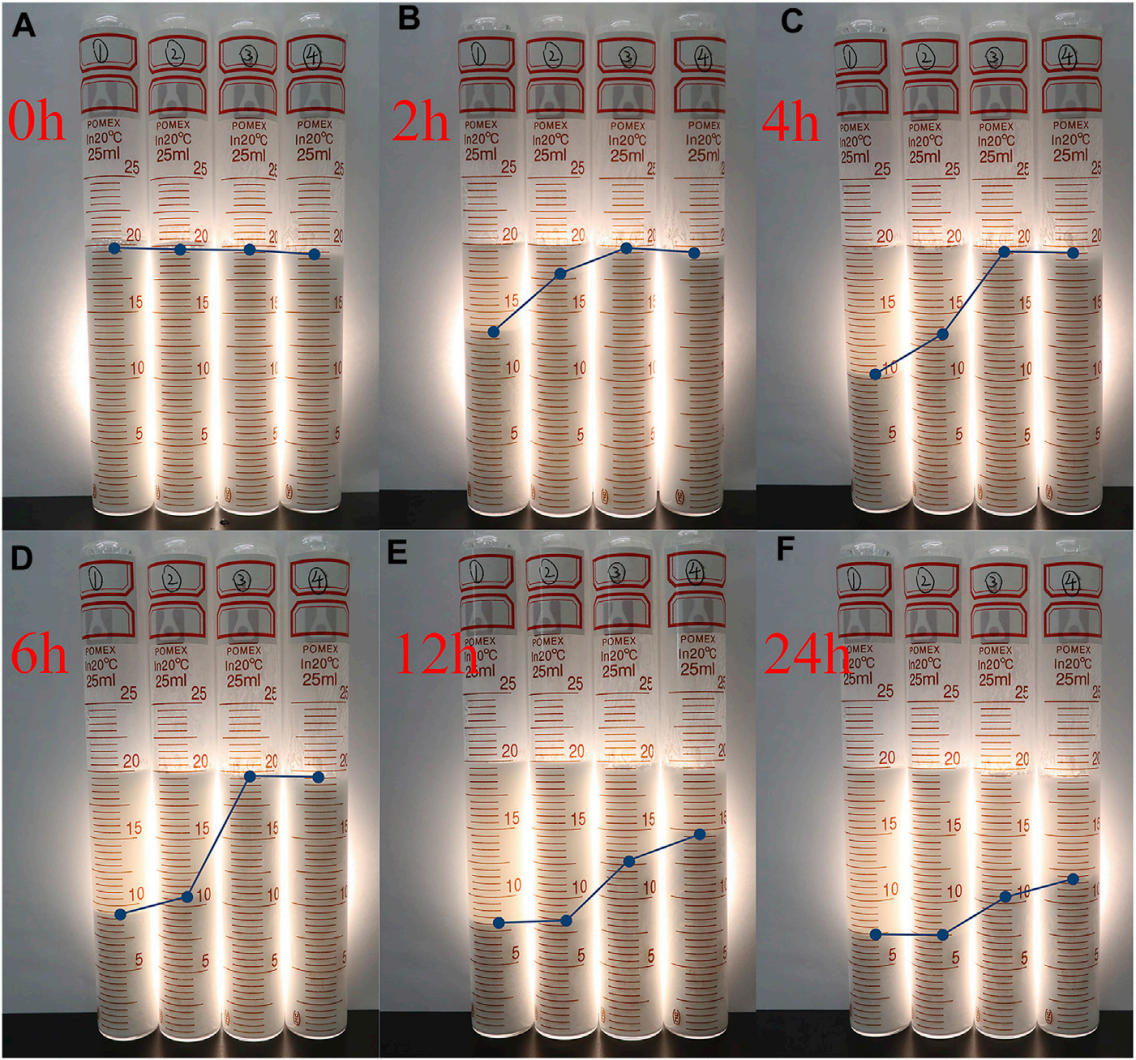
Influence of MPL on the stability of water-in-oil emulsion. The MPL concentrations in each photo are 0, 1, 2 and 3% from left to right. The standing time is 0 h, 2 h, 4 h, 6 h, 12 h, 24 h for Photo **(A–F)**, respectively.

The effect of the MPL on the stability of the inverse emulsion with an oil-water ratio of 80:20 was studied through observations. The experimental results are shown in Figure 6. For the emulsion without the MPL, serious sedimentation occurred within 2 h, and a large amount of oil was precipitated in the upper layer of the emulsion (Figure 6B). The sedimentation was more pronounced at 6 h, and the volume of the upper layer oil accounted for 55.0% of the total volume. However, the emulsions containing 2 and 3% MPL were not layered within 6 h, and the addition of 1% MPL only slightly improved the stability of the emulsion. It should be noted the solid content of the synthesized MPL was 30%. Therefore, the emulsion containing 1% MPL had a lower nanoparticle concentration (0.3%). Besides, after the addition of 2 and 3% MPL, the emulsions appeared to settle when left to stand for 12 and 24 h. But the volume of the upper oil layer was lower than those of the emulsions without MPL. Therefore, the MPL had a significantly impact on improving the stability of the emulsion.

**FIGURE 7:**
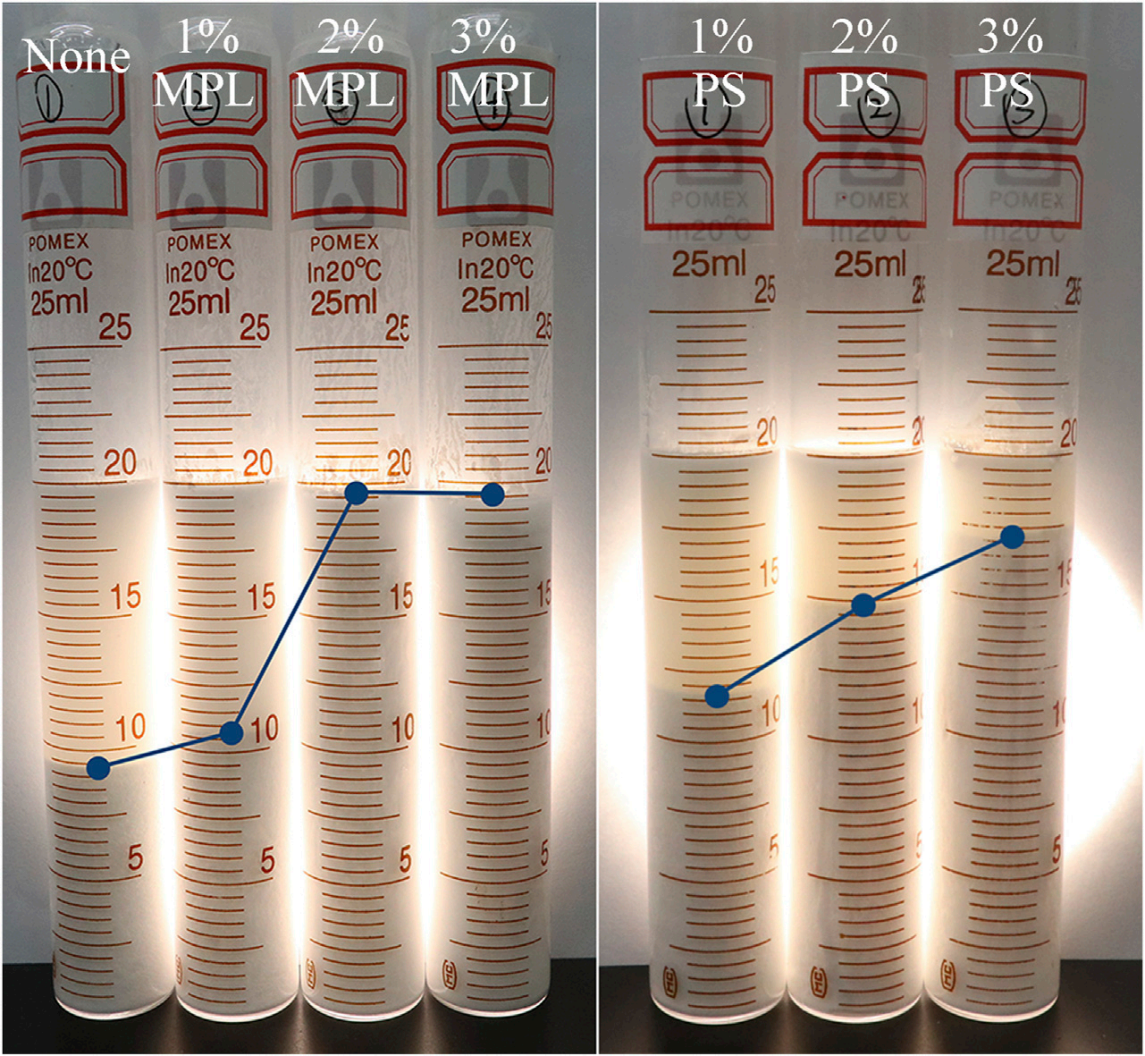
The influence of PS and MPL on the stability of the emulsion. The standing time is 6 h.

Comparative experiment was conducted with polystyrene nanospheres (PS). Figure 7 shows the state of the emulsion with different concentration of MPL and PS after standing for 6 h. PS was also beneficial to improve stability of the invert emulsion. With the increase of PS addition, the volume of top oil gradually decreased. The emulsion stabilization effect of PS was slightly better than that of MPL at the concentration of 1%. But when the concentrations were 2 and 3%,.MPL performed much better than PS.

The electrical stability is a measure of how well the water is emulsified in the continuous oil phase. For the same emulsion, a higher demulsification voltage is indicative of a higher stability. As is shown in Table 1, PS almost have no effect on demulsification voltage. When the MPL was added to the emulsion with an oil-water ratio of 80:20, the ES value of the emulsion increased from 283 to 553 V as the concentration of the MPL increased from 0 to 3%. Organoclay is an important component of OBFs, and it is usually used as a rheology modifier. Adding 2% organoclay to the emulsion can greatly improve the ES value of the emulsion. When the MPL was added to the emulsion containing organoclay, the ES value of the emulsion continued to increase. When MPL concentration was 2%, ES values reached the maximum value of the ES meter (2,047 V). Therefore, the addition of the MPL can also effectively improve the electrical stability of OBFs.

**TABLE 1:** The influence of polystyrene nanospheres and MPL on demulsification voltage.

Concentration (%)	Electrical stability (V)		
	Emulsion + PS	Emulsion + MPL	Emulsion + 2% Organoclay + MPL
0	283	283	1467
0.5	276	310	1690
1.0	287	342	2007
2.0	281	429	2047
3.0	296	553	2047

**FIGURE 8:**
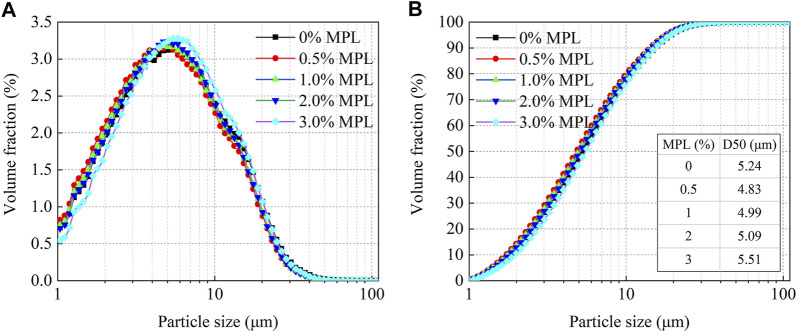
**(A)** Differential particle size distribution and **(B)** cumulative particle size distribution of water-in-oil emulsions with different MPL concentrations.

To analyze the emulsion stabilization mechanism of the MPL, its effect on the particle sizes of the water-in-oil emulsions was investigated. The experimental results are shown in Figure 8. The particle size of the emulsion was about 5 μm, and the addition of the MPL had little effect on the particle size of the emulsion (within the error range). Therefore, the MPL did not increase emulsion’s stability through the reduction of the particle size.

### Plugging Performance

The effect of the MPL on the plugging of microporous membranes with different pore sizes in two mediums (i.e., mineral oil and inverse emulsion) was evaluated *via* filtration loss experiments.

**FIGURE 9:**
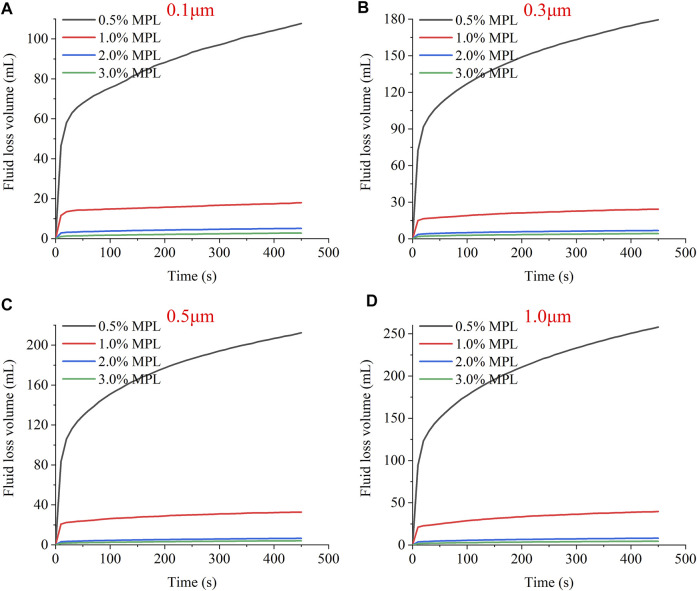
The fluid loss volume *vs*. time curves for MPL oil dispersions for microporous membranes with different pore sizes: **(A)** 0.1 μm, **(B)** 0.3 μm, **(C)** 0.5 μm, and **(D)** 1.0 μm.

Figures 9A–D present the curves of the filter loss of the MPL oil dispersion with time. The experiments revealed that white oil quickly passed through the microporous membranes of all pore sizes and was completely filtered through in a short time. However, after adding 0.5% MPL, the filtration rate of the white oil decreased significantly, and the filtration was not complete after 7.5 min. For all of the microporous membranes with different pore sizes, as the MPL concentration increased, the filtration loss of the white oil dispersion consistently decreased. For a concentration of 2%, over a 7.5 min (450 s) duration, the losses through the 0.1, 0.3, 0.5, and 1.0 μm pore size filters were 5.1, 6.8, 6.5, and 8.1 ml, respectively. This demonstrates that the addition of the MPL to the white oil improved the plugging effect. At concentrations of > 2%, as the MPL concentration increased, a less noticeable decrease in the filtration loss occurred.

**FIGURE 10:**
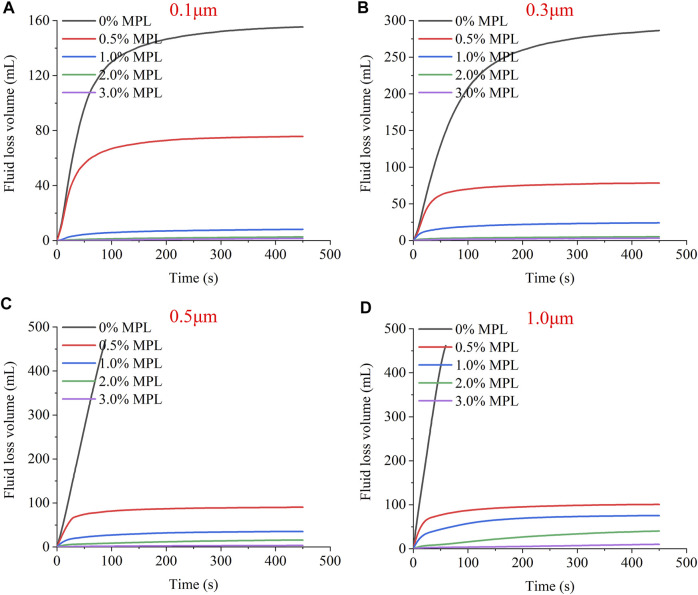
The fluid loss volume *vs*. time curves for MPL dispersions in emulsion for microporous membranes with different pore sizes: **(A)** 0.1 μm, **(B)** 0.3 μm, **(C)** 0.5 μm, and **(D)** 1.0 μm.

Figures 10A–D are plots of the filter loss versus time for the MPL dispersion in an emulsion. For the 0.1 and 0.3 μm microporous membranes, the emulsion without MPL was not completely filtered. When the pore size was ≥ 0.5 μm, the emulsion was completely filtered within 100 s, which indicates that the plugging effect was poor for the water-in-oil emulsion. After the white oil was combined with water to form an emulsion, the viscosity of the liquid increased and the internal phase droplets had a certain size, which produced the slight plugging effect. After the MPL was added, the filtration loss decreased significantly, further demonstrating that the MPL has a good plugging effect in the water-in-oil emulsion.

Regarding the trends of the filter loss curves in Figures 9, 10, all of the curves have large slopes in the initial stage, and the filter loss increases rapidly with time. However, after the initial stage, the slopes of the filter loss curves gradually decrease. For the MPL oil dispersion, when the MPL concentration is ≥ 1.0%, the filter loss curve tends to flatten in a short period of time (<20 s), and the filter loss slowly increases or does not increase at all. For the MPL dispersion in an emulsion, most of the slopes also ceased to increase within 100 s. This shows that the MPL completely plugged 0.1–1.0 μm microporous membranes in a short time; providing further evidence that the MPL has a favorable plugging performance.

The micro-scale pores and fractures in shale formations are widely distributed and occur at various scales. Achieving more than adequate plugging of micropores and fractures of different scales poses a particular challenge when rigid particles are involved. According to the temporary plugging theory, the particle size of a rigid plugging agent should match the sizes of the microscale pores and fractures in the formation in order to have a good performance. The current plugging theories mainly include: the 1/3 bridging rule proposed by Abrams (Abrams, 1977), the 2/3 bridging rule proposed by Luo and Luo (Luo and Luo, 1992), and the ideal packing theory proposed by Dick et al. (Dick et al., 2000). However, soft plugging agents can adapt to wide-scale pores and fractures by deforming themselves.

During the drilling process, a layer of oil film will inevitably be adsorbed unto the surfaces of the rock pores and fractures due to the filtration of OBFs. This oil film layer weakens the adhesion between the plugging material and the surface of the rock matrix. As such, the plugging materials commonly used in OBFs do not easily adhere to the surfaces of pores and fractures, resulting in ineffective plugging mechanisms. Long-chain acrylate monomers are introduced in the synthesis process, so the MPL nanoparticles are a sticky material that can strongly adhere to rock surfaces. This property is also conducive to an improved plugging effect.

## Conclusions

A modified polystyrene latex (MPL) with a solid content of 30% was synthesized *via* emulsion polymerization. The D50 of the MPL nanoparticles is 233 nm, and the initial thermal decomposition temperature is 363°C. The three-phase contact angle of the MPL film is 103.5°. After adding the MPL, the sedimentation stability and electrical stability of the water-in-oil emulsion were greatly improved. The MPL itself had little effect on the particle size of the emulsion, indicating that the improvement of the stability was not achieved by reducing the particle size of the emulsion. The MPL can greatly reduce filtration loss in both white oil and inverse emulsions, and MPL reduced the filtration loss through 0.1–1.0 μm microporous membranes to within 10 ml when the concentration was 3%. The MPL has a strong adaptability to micro pores of different sizes, and it can completely plug microporous membranes with pore sizes of 0.1–1.0 μm. Thus, MPL could improve emulsion stability and plugging performance of nanopores in invert emulsion drilling fluids.

## Data Availability

The original contributions presented in the study are included in the article/Supplementary material, further inquiries can be directed to the corresponding author.
